# Motivation, stress, recovery, and physical activity of teachers: discoveries with the Reiss Motivation Profile^®^ and the Firstbeat^®^ measurement

**DOI:** 10.1007/s10389-023-01828-1

**Published:** 2023-01-26

**Authors:** Pipsa P. A. Tuominen, Päivi Mayor

**Affiliations:** 1 Tampere University of Applied Sciences, Social Services and Health Care, Physiotherapy degree program, Kuntokatu 3, P1-13, 33520 Tampere, Finland; 2 Tampere University of Applied Sciences, Business and Media, International Business, Master’s in Educational Leadership degree program, Tampere, Finland

**Keywords:** Motivation, Autonomous nervous system, Relaxation, Exercise, Inactivity

## Abstract

**Aim:**

Teaching is known as a high-stress occupation. Managing fatigue and promoting work engagement, teachers’ expectations and perceptions can impact work-related stress–recovery–balance and physical activity levels. The main objective of the current study was to investigate the relationship between intrinsic motivational factors and the autonomic nervous system, such as heart rate and heart rate variability regulation, sleep, and physical activity levels.

**Methods:**

The research included 66 primary and high school teachers in Tampere, Finland, in 2020–2021. The Reiss Motivation Profile® (RMP) was selected as an instrument to provide a practical approach to understanding people’s intrinsic motivators and the effects of those on the Firstbeat®-measured heart rate and heart rate variability, and accelerometry. Stepwise multiple linear regression models were used to assess whether the RMP motives reflected Firstbeat®-measured outcomes.

**Results:**

Motives such as Acceptance, Eating, Family, Physical Activity, Power, Tranquillity, and Vengeance were found to have a relationship with Firstbeat®-measured outcomes. An increase in the Physical Activity motive was related to lower stress and less light physical activity. Furthermore, an increase in the Physical Activity motive showed a relationship with better recovery and a higher amount of vigorous physical activity. However, the statistical analysis of heart rate and heart rate variability showed only low explanatory power (R^2^ = 0.111–0.140) for stress, recovery, and sleep. On the contrary, the explanatory power of measurements related to physical activity and sedentary behavior was higher (R^2^ = 0.171–0.298). In addition, the need for Acceptance as an important intrinsic motive that may prevent people from vigorous physical activity deserves further research.

**Conclusion:**

The results indicated that there are several factors affecting the autonomous nervous system but also behavior. The basic desires explained sedentary and physical activity behavior better than the functioning of the autonomic nervous system. The 16 life motives can serve as a valuable theory for understanding better how to encourage people to have healthier habits.

## Background

Teaching is known as a high-stress occupation. To manage fatigue and promote the capacity for work engagement, teachers’ expectations and perceptions can impact work-related stress-recovery-balance and levels of physical activity (PA). In earlier studies, time spent on work-related tasks outside of formal working hours was related to increased fatigue (Garrick et al. 2018). Further, female teachers have reported lower perceived health and more occupational stress than their male colleagues (Bogaert et al. 2014). In contrast, good sleep quality and time spent socializing outside of work were related to reduced fatigue (Garrick et al. 2018). Also, feelings of detachment, relaxation, meaning, and affiliation were linked to higher life satisfaction (Virtanen et al. 2020).

The effect of PA and sedentary habits on perceived health and indirectly on job satisfaction have been largely studied. The amount of PA has also been connected to workability. For example, higher participation in leisure-time PA has been associated with positive perceived health. Bogaert et al. (2014) also concluded that teachers who exercise more during their leisure time might be more resistant to physical and mental health problems. In addition, older teachers have been found to benefit more from control and mastery during their leisure time compared to younger teachers, who benefited more from relaxation (Virtanen et al. 2020). Further, discrete emotions have been found to represent motivational constructs that may help explain subjective experiences and volitional moderate to vigorous PA. In addition, enjoyment was a predictor of PA behavior and a preventive to sedentary time (Simonton 2021).

### Firstbeat® lifestyle assessment

Concerning daily life, previous studies have widely studied a balance between physical activity (PA), stress, and recovery (Föhr et al. 2015, 2016, 2017; Pantzar et al. 2017). The balance can be studied since the autonomic nervous system reacts and causes heart rate (HR) and heart rate variability (HRV) changes. These changes can be measured by an HR monitor, such as the Firstbeat® (FB) device. When the sympathetic nervous system is effectively activated and blood pressure, heart rate, and respiration increase, the body’s energy production and performance ability increase. This is known as a stress reaction. In turn, when the parasympathetic nervous system is activated, HR and respiration slow down, and HRV increases, causing a recovery reaction.

In previous studies, subjective stress has been found to associate directly with device-measured stress, i.e., smaller HRV, and inversely with recovery, i.e., larger HRV (Föhr et al. 2015). Furthermore, daily stress is highest in the mornings and around eight o’clock in the evenings on average (Pantzar et al. 2017).

The Firstbeat® device also includes a tri-axial accelerometer that can measure PA levels and the amount of sedentary behavior (SB). Higher PA levels have been associated with lower subjective stress in cross-sectional settings (Föhr et al. 2015) and positively affect subjective stress changes in the long term (Föhr et al. 2016, 2017). In addition, better recovery from stress and a strong sense of meaningfulness have been predicted to increase PA (Mutikainen et al. 2015).

### Research on motivation according to Steven Reiss

In this study, we used a motivation theory of the 16 basic desires or life motives of Professor Steven Reiss (Reiss and Havercamp 1998, 2005; Reiss 2008, 2013a). In 1998 Steven Reiss and Susan Havercamp conducted extensive empirical studies with thousands of participants from various nations and a series of factor-analytic studies, eventually resulting in 16 distinct universal reinforcements for behavior. The 16 life motives (desires, values, or goals) drive all human beings and motivate human behavior. These needs are universal, but to what extent they are motivated by them and how much they want them is highly individual (Havercamp and Reiss 2003). Based on his research results, Reiss argued that all motivation is intrinsic in nature and people seek to fulfill one or more of the 16 basic desires with their behavior. According to Reiss (2013a), the intensity of motivation is a continuum, and also rewards (often linked to the term extrinsic motivation by Deci and Ryan, e.g., 2008) may increase motivation positively when seen as signs of achievement, recognition, and success (Reiss 2013a). (For more information about Reiss’ research and arguments against the division of intrinsic and extrinsic motivation, see Reiss 2013a.)

The 16 basic desires, values, or goals are:

- Acceptance, the desire for positive self-regards
- Curiosity, the desire for understanding
- Eating, the desire for food
- Family, the desire to raise children and spend time with siblings
- Honor, the desire for upright character
- Idealism, the desire for social justice
- Independence, the desire for self-reliance
- Order, the desire to be organized and clean
- Physical activity, the desire for muscle exercise
- Power, the desire for influence and leadership
- Romance or beauty, the desire for sex or beauty
- Saving, the desire to collect
- Social contact, the desire for peer companionship
- Status, the desire for respect based on social standing
- Tranquillity, the desire to be free of anxiety and pain
- Vengeance, the desire to confront those who offend (Reiss 2008)

The individual intensity of the 16 basic desires can be measured with the Reiss Motivation Profile® (RMP). This valid and reliable standardized assessment tool has been used in many areas of life, such as business coaching, leadership development, sports coaching, marriage counseling, and study counseling. In addition, the RMP has been tested and validated by several independent and peer-reviewed research studies (e.g., Reiss et al. 2001; Havercamp and Reiss 2003; Reiss and Havercamp 1998; Reiss 2004; Reiss and Havercamp 2005; Reiss 2005) and the practical value presented in an increasing number of studies and publications, for example, in the areas of psychology and leadership (Reiss and Wiltz 2004; Olsen and Chapin 2007; Reiss 2008; Mengel 2012; Mayor and Risku 2015; Chudzicka-Czupala and Basek 2019; Morawski and Jabłonowska-Luba 2021).

The Reiss Motivation Profile® was selected as an instrument for this study because of its scientific basis on large empirical studies and its strong validity and reliability (Reiss and Havercamp 1998; Havercamp and Reiss 2003). Reliability studies showed that the RMP results are likely to remain stable and consistent over time (Reiss and Havercamp 1998) and the study related to the internal consistency of the RMP was considered to be good (Havercamp and Reiss 2003). RMP also provides a practical approach to understanding people’s intrinsic motivators (Jones 2021).

To our knowledge, there are no studies related to teachers’ motives and device-measured stress, recovery, and PA. Further, earlier research on PA and sedentary behaviors, and personal desires explores the efficacy of exercise and has been often related to maladaptive behaviors (Stults-Kolehmainen et al. 2020). Therefore, based on the theoretical assumption that motives affect activities in many areas of life and, thus, impact the autonomic nervous system, the objective of the current study was to find what motives could be seen in the measurements based on HR, HRV, and acceleration signals (i.e., PA and SB).

## Methods

### Study design and participants

This is a cross-sectional study with an explorative approach. We were interested in discovering what relationships and connections could be found between how individuals prioritize the 16 basic desires and how those could be identified in the Firstbeat® measurement. Since no previous study has been done about the 16 basic desires and the FB measurement results, all the motives were taken into consideration in the study. The RMP version used in this study was the RMP for Business in which the Romance scale has been replaced with the also validated Beauty scale.

The participants for the current study were 66 primary and high school teachers in the municipality of Tampere, Pirkanmaa region in Finland. They participated in the European Social Fund-funded research project called the Sustainable Brain Health. Tampere University of Applied Sciences coordinated the project. The city of Tampere appointed the schools to participate in the Sustainable Brain Health Project, and each school asked voluntary teachers to participate.

Taking the RMP and the FB measurement was entirely voluntary. Study information was given to the participants both orally and in writing before the data collection. All the participants provided written consent about the data collection and use for research purposes. Due to the voluntary participation of healthy adults, ethics committee approval was not needed (TENK 2019).

The main objectives of the Sustainable Brain Health Project were to identify and improve the target group’s (such as teachers, nurses, and IT workers) health and well-being, reinforce self-direction and self-leadership, and establish procedures and tools for sustainable brain health (Sustainable Brain Health n.d.). This paper reports the results of the relationship between teachers’ RMP and FB measurements.

The RMP and FB measurements were conducted between November 2020 and January 2021. During this period, the schools of the participants had contact teaching; the lockdown due to the Covid-19 situation occurred from March to May 2020. In addition, there were additional safety measures such as wearing masks, separating classes and groups from each other, and encouraging everybody to keep a safe distance from other people.

### Measurements and outcomes

The RMP was used to measure how individuals prioritize the 16 basic desires or motives. The RMP is a self-report questionnaire comprising 128 items, eight items for each of the 16 scales. Responding to the electronic questionnaire takes about 15–30 minutes to complete. The results are illustrated as a graph in the form of standard distribution comparing the results to a norm, based on the country of residence, in this case, Finland, and the gender of the respondents. The resulting scores for a scale range from –2.00 to +2.00, whereby the minus results indicate a low need for that particular motive, and the plus score indicates a high need. If the result is between ­0.84 and +0.84, the motivation is situational, and the result is similar to approximately 60% of the norm group.

The FB Bodyguard 2 device was used to measure HR, HRV, and tri-dimensional acceleration signal. The device electrodes were attached directly to participants’ chests at two points. The device was used for three continuous days and nights, except during water sports and showering. The data for stress, recovery, sleep, SB, and light to vigorous PA were distinguished with the FB analysis program and exported to SPSS statistical software for analysis.

For study purposes, RMP motives and baseline descriptive statistics, such as age, gender, marital status, having children or nursing for loved ones, teaching experience, permanent employment, superior position, and telecommuting more than half were used to explain the results of FB measurements.

### Statistical methods

Statistical analyses were performed using SPSS statistical software version 27 (IBM, Armonk, NY, USA). RMP motives and baseline descriptive statistics were used to characterize participants’ FB measurement results. These characteristics were reported as frequencies and percentages for categorical variables and as means and standard deviations (SD) for continuous variables since they were normally distributed. The normal distribution n test was checked with a histogram and a Q-Q plot. The linearity assumption between the independent and dependent variables before the modeling and predicted values after the modeling and homoscedasticity of residuals were tested with scatter plots. Finally, multicollinearity was examined using the correlation matrix.

As a first step, the univariate prescreening of variables was done to find variables that were the best to explain outcomes. The second step was the use of stepwise multiple linear regression models to assess whether the RMP motives reflected FB-measured outcomes, i.e., stepwise selection of variables. Characteristics of participants were also used as potential predictor variables. Specifically, we were interested in seeing which variables were the strongest, i.e., significantly explaining the variation in the response variable of all the 16+ variables. Finally, the maximum number of selected explanatory variables was set to four because of the number of participants, representing around 15 participants per each explanatory variable (Green 1991). Gender and teaching experience were forced into the models of stress and recovery, and Family motive into the model of moderate PA. For each outcome, the model with the highest adjusted r square was chosen to report even if the forced variables did not show statistical significance.

## Results

### Characteristics of participants

All participants had a master’s degree at the university level. They worked in primary schools teaching pupils from ages 6 to 12 or in high schools teaching pupils from ages 13 to 16. Further characteristics of the participants are presented in Table 1 below.

**Table 1:** Characteristics of participants

	n	Mean (SD) or n (%)
Age in 2020	66	46.4 (9.1)
Gender, female	66	61 (92.0)
Married/cohabited	66	57 (86.4)
Having children in the Family	66	39 (59.1)
Nursing for loved ones	66	16 (24.2)
Teaching experience, years	66	11.4 (9.2)
Employment, permanent	66	60 (90.9)
Superior position	66	2 (3.0)
Telecommuting, more than half	66	5 (7.6)

### Outcome characteristics

**Fig. 1:**
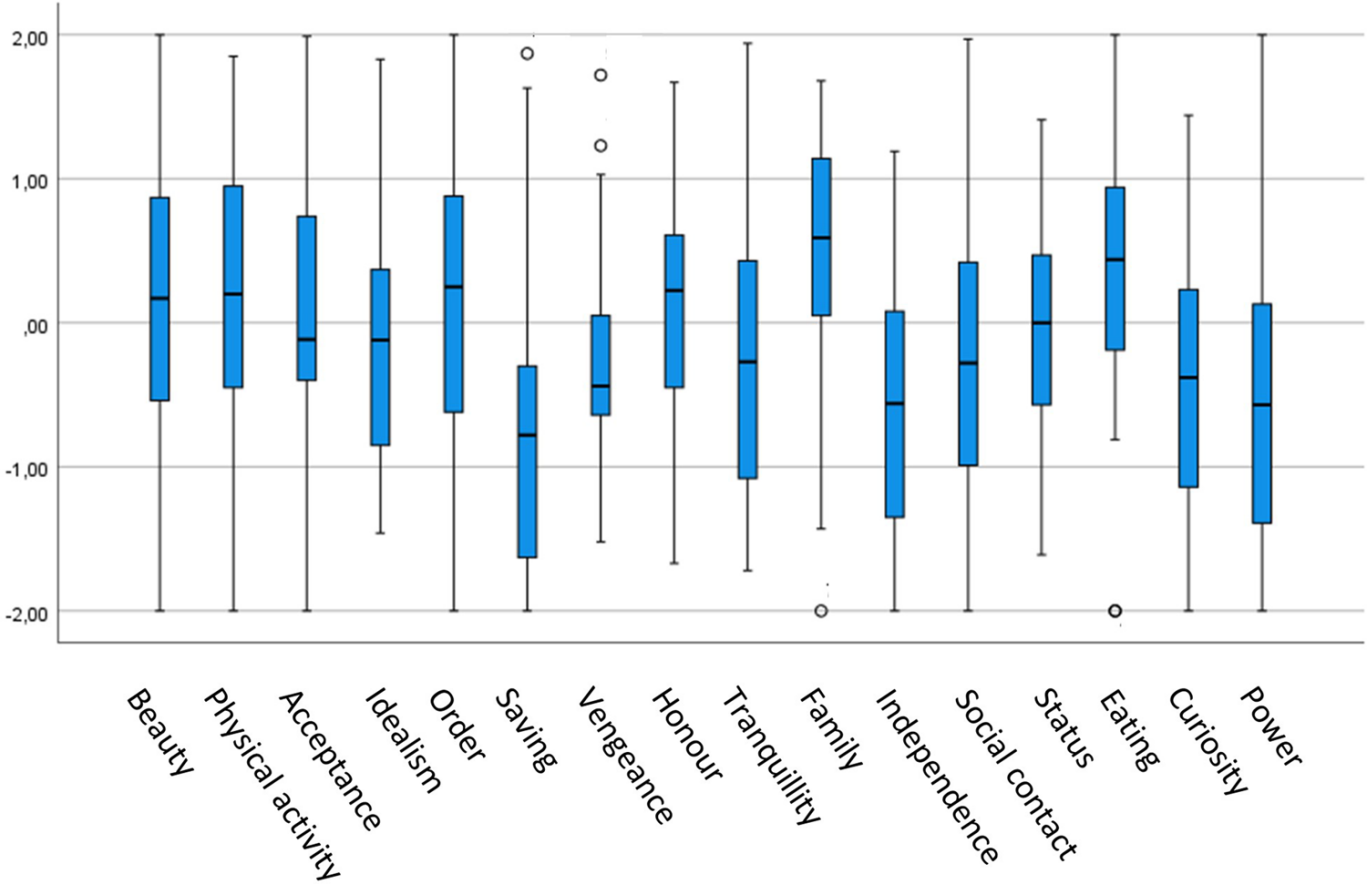
The data from 16 RMP basic desires. Whiskers show the smallest and the largest values of each data set. The boxes show the second and third quartiles. The median is shown as a line in the center of the box. Outliers are shown as small circles

The box and whisker chart (Fig. 1) shows the data related to RMP basic desires. Means, standard deviations, and Pearson correlations for the dependent and independent variables are presented in Table 2. As device-measured variables, stress, recovery, and sleep are based on HR and HRV, and proportions are related to the total measurement time. Further, proportions of SB and light to vigorous PA are based on a built-in tri-axial accelerometry, HR, and HRV.

**Table 2:** Means, standard deviations, and Pearson correlations among the dependent and all independent variables

Variable		Mean (SD)	1	2	3	4	5
Stress proportion		55.9 (12.0)	–	–0.303^**^	0.211^*^	0.209^*^	
RMP physical activity motive		0.22 (0.98)		–	–0.010	–0.285^*^	
Gender, female (%)		92.0			–	–0.015	
Teaching experience (years)		11.4 (9.2)				–	
Recovery proportion		22.4 (9.1)	–	0.333^**^	–0.233^*^	–0.212^*^	
RMP physical activity motive		0.22 (0.98)		–	–0.010	–0.285^*^	
Gender, female (%)		92.0			–	–0.015	
Teaching experience (years)		11.4 (9.2)				–	
Sleep proportion		21.1 (8.1)	–	–0.248^*^	0.284^*^		
RMP acceptance		0.05 (0.94)		–	–0.009		
Superior position (%)		3.0			–		
Sedentary behavior proportion		58.7 (12.3)	–	0.221^*^	–0.147	–0.368^**^	–0.300^**^
RMP power		–0.55 (0.95)		–	0.138	–0.081	0.190^*^
RMP eating		0.42 (0.87)			–	–0.326^**^	0.106
Age in 2020		46.4 (9.1)				–	0.118
Superior position (%)		3.0					–
Light physical activity proportion		14.0 (6.8)	–	–0.417^**^	0.167	0.360^**^	–0.350^**^
RMP physical activity motive		0.22 (0.98)		–	–0.088	–0.285^*^	0.252^*^
RMP tranquility		–0.23 (0.88)			–	–0.278*	0.023
Teaching experience (years)		11.4 (9.2)				–	–0.172
Nursing for loved ones (%)		23.9					–
Moderate physical activity proportion		2.7 (1.7)	–	–0.283^*^	0.327^**^	–0.236^*^	0.227^*^
RMP family		0.50 (0.79)		–	–0.114	0.134	0.063
Teaching experience (years)		11.4 (9.2)			–	0.329^**^	–0.015
Permanent employment (%)		90.9				–	–0.091
Gender, female (%)		92.0					–
Vigorous physical activity proportion		2.8 (2.1)	–	0.286^*^	–0.267^*^	0.159	
RMP physical activity motive		0.22 (0.98)		–	–0.091	–0.019	
RMP acceptance		0.05 (0.94)			–	0.338^**^	
RMP vengeance		–0.30 (0.63)				–	

^*^p < 0.05, ^**^p < 0.01

### Relationship between the Reiss Motivation Profile® motives and Firstbeat® measurements

Starting with 16 motives and nine background characteristics that might theoretically be good predictors of FB-measured outcomes, stepwise linear regression models were able to reduce them to 2–4 per outcome. RMP motives such as Acceptance, Eating, Family, Physical Activity, Power, Tranquillity, and Vengeance were found to have a relationship with FB-measured outcomes. Explanatory factors (standardized β-coefficient with p-value) and model fit statistics (adjusted R^2^ and F-statistics with p-value) are presented in Table 3.

**Table 3:** A relationship between RMP motives and FB measurements in linear regression models

	Explanatory factors		Model fit statistics	
	Standardized β-coefficient	Significance (p-value)	Adjusted R^2^	F-statistics with significance (p-value)
Stress proportion			0.111	F(3, 62), 3.716, 0.016
Physical activity motive	–0.261	0.036		
Teaching experience	0.138	0.263		
Gender (female)	0.211	0.076		
Recovery proportion			0.140	F(3, 62), 4.525, 0.006
Physical activity motive	0.293	0.018		
Teaching experience	–0.132	0.275		
Gender (female)	–0.232	0.048		
Sleep proportion			0.113	F(2, 63), 5.154, 0.008
Acceptance motive	–0.245	0.040		
Superior position	0.281	0.019		
Sedentary behavior proportion			0.298	F(4, 61), 7.892, < 0.001
Power motive	0.288	0.010		
Eating motive	–0.291	0.012		
Age	–0.407	0.001		
Superior position	–0.281	0.012		
Light physical activity proportion			0.297	F(4, 61), 7.871, < 0.001
Physical activity motive	–0.247	0.029		
Tranquillity motive	0.238	0.032		
Teaching experience	0.314	0.008		
Nursing for loved ones	–0.241	0.028		
Moderate physical activity proportion			0.275	F(4, 61), 7.172, < 0.001
Family motive	–0.206	0.062		
Teaching experience	0.414	0.001		
Permanent employment	–0.325	0.006		
Gender (female)	0.217	0.027		
Vigorous physical activity proportion			0.171	F(3, 62), 5.459, 0.002
Physical activity motive	0.260	0.025		
Acceptance motive	–0.337	0.007		
Vengeance motive	0.278	0.024		

P-value < 0.05 indicates a statistical significance

Physical Activity motive, gender, and teaching experience explained 11.1% of FB measured stress and 14.0% of recovery proportions. Participants’ stress proportion decreased for each plus score in Physical Activity motive and increased for each year of teaching experience. Further, female participants showed a higher stress proportion compared to males. However, only the Physical Activity motive was a significant predictor for stress proportion. In turn, participants’ recovery proportion increased for each additional score in the Physical Activity motive and decreased with teaching experience. In addition, females had a lower recovery proportion than males. Here, the Physical Activity motive and gender were significant predictors for recovery proportion.

Acceptance motive and being in a superior position explained 11.3% of FB measured sleep proportion. Sleep proportion decreased for each additional score in the Acceptance motive. Further, participants in a superior position slept more than others. Both the Acceptance motive and a superior position were significant predictors of sleep.

Power and Eating motives, age, and a superior position explained 29.8% of FB measured SB proportion. SB increased for each additional score in the Power motive and decreased for the Eating motive. Also, each additional year of age decreased SB. Finally, participants in a superior position had less SB than others. All of these variables were significant predictors for SB.

Physical Activity and Tranquillity motives, teaching experience, and nursing for loved ones explained 29.7% of FB measured light PA proportion. Light PA decreased for each additional score in the Physical Activity motive and increased for the Tranquillity motive. Also, each additional year of teaching experience increased light PA. Finally, participants who nursed for loved ones had less light PA than others. All of these variables were significant predictors for light PA.

Family motive, teaching experience, permanent employment, and female gender explained 27.5% of FB measured moderate PA proportion. Moderate PA decreased for each additional score in Family motive. Each additional year of teaching experience increased moderate PA. Further, participants with permanent employment showed less moderate PA than others, and females had a higher proportion of moderate PA than males. The Family motive was nearly significant, and teaching experience, permanent employment, and gender were significant predictors for moderate PA.

Physical activity motive, Acceptance motive, and Vengeance motive explained 17.1% of FB measured vigorous PA proportion. Vigorous PA increased for each additional score in the Physical Activity motive and Vengeance motive and decreased for each additional score in the Acceptance motive. All of these variables were significant predictors for vigorous PA.

## Discussion

In the current study, we had the theoretical assumption that motives affect activities in many areas of life. Thus, both intentions and actions impact the autonomic nervous system. We explored what were the motives that could affect the measurements based on HR, HRV, and acceleration signal (i.e., PA and SB).

The RMP motives such as Acceptance, Eating, Family, Physical Activity, Power, Tranquillity, and Vengeance were found to have a relationship with FB-measured outcomes. The *adjusted* R-squared compares the explanatory power of regression models that contain different numbers of predictors. However, in this study, the explanatory power related to HR and HRV showed lower explanatory power than accelerometer measurements did.

### Stress, recovery, and sleep proportion

The explanatory power of RMP motives and background information on stress, recovery, and sleep proportion were between 11% and 14%. In this study, 2–4 explanatory variables, a combination that offered the highest explanatory power, were selected for each of the response variables due to the number of participants, based on the work by Green (1991). Since there were altogether 16 motives and further background information, it seems obvious that a larger population should have been studied for the higher explanatory power (i.e., the number of explanatory variables could have been higher if there had been more participants). However, participation in the study was completely voluntary, and 66 teachers selected both RMP and FB measurements.

Another explanation for the low explanatory power is related to Reiss’s (2004) theory, which conceptualized human behavior as a function of instincts, drives, needs, and tensions. However, there might be some other factors than motives that influence these aforementioned three aspects (stress, recovery, and sleep), such as physical inactivity, overweight, workload, and work-related stress (Föhr et al. 2016).

According to our results, stress decreased when the Physical Activity motive increased. We assume that people who have a higher Physical Activity motive would actually move more and with a higher power, and thus, they might experience less stress and have more rest. Similarly, when the Physical Activity motive increases, the recovery proportion also increases. Therefore, we think that those with a high training load would also need and take more time for recovery. These results are in line with studies explaining the positive influence of moving and sports. For example, Föhr et al. (2017) concluded that high PA and device-assessed low stress (i.e., larger HRV) and good recovery positively affect subjective stress changes. Wahl et al. (2020) found that exercise intensity tolerance directly affected perceived stress and recovery. In addition, the accumulation of stress responses was found more predictive of subsequent PA than current stress reactivity or recovery responses (Almeida et al. 2020).

Gender and teaching experience were forced to regression models of stress and recovery since the explanatory power was higher with these. Our finding related to women having greater stress proportion to men is in line with Klassen and Chiu (2010). Further, we assume that increasing experience in a job may help reduce stress and find ways to recover, but that does not seem to be the case in this population. The teaching experience was around 11 years on average, and thus, our result contradicts Klassen and Chiu (2010) who found that from early career to mid-career self-efficacy and, thus, possibilities for recovery, increased. This could be because of the participants’ average age of 46 years with possibly limited possibilities to recover on leisure time and more family-related responsibilities.

Related to sleep proportion, when the need for Acceptance increased, the amount of sleep decreased. Based on Reiss (2008), individuals who have an increased demand for Acceptance do not want to be criticized or rejected. Therefore, they might have less self-confidence than those with a low need for Acceptance. They may easily blame themselves and worry that their performance may be evaluated as inferior sometimes leading to working longer and harder to please others (Reiss 2008, 2013b). Therefore, the influence of the need for Acceptance and the diminishing amount of sleep is understandable: their sleep may be influenced by worrying if they performed well enough and the sheer amount of work hours and effort driven by the high need for Acceptance by others.

Furthermore, in this population, when a person was in a superior position, they slept longer than others. This is somewhat surprising because one might assume that managers and leaders may be more stressed than others, and this can also be seen in their sleep length. One explanation may be found in the low need for Acceptance. This is only speculation, but people with a low need for Acceptance are often more likely to be asked to take a leadership role since they behave more confidently than others. However, only two individuals (3%) were in a superior position in this population, and thus, these results are not generalizable.

### Sedentary behavior and light to vigorous physical activity

The explanatory power of RMP motives and background information on SB and light to vigorous PA was approximately 17% to 30%, the lowest for vigorous PA and the highest for SB. In the current study, the motivation theory by Reiss did not fully explain dynamic changes in health-related behaviors. Based on the paper by Stults-Kolehmainen et al. (2020), affectively charged motivation states that change quickly may better explain PA behavior from one moment to the next than longer-lasting desires, values, or goals.

Related to SB, we found that SB increased when the need for Power increased. According to Reiss (1998, 2008), people with a high need for Power tend to work a lot and influence others in many places and situations. If the majority of the work takes place at a computer, that may increase the amount of sitting and other SB.

Also, when the Eating motive increased, SB decreased. There could be many explanations for this: During lunch breaks at school, many teachers have simultaneous supervisory roles, including moving around in the canteen to ensure that children eat, too. Also, people who eat regularly supported by this motive may have more energy to move. The latter is in line with Stuntz et al. (2017), who found that healthy eating habits were associated with higher leisure-time PA mediated through psychological variables. The higher levels of PA decrease the amount of SB, at least if the intensity of PA stays light or moderate (i.e., in sub-maximal levels of intensity).

Further, with higher age, SB decreased. This may be related to the increased awareness of and interest in improving health and reducing stress. This assumption is in line with Loosveldt (2015), who found that paying attention and being aware of the harmful effects of SB lead to small positive changes in everyday routine, such as increasing non-exercise physical activities.

In addition, being in a superior position decreased SB. We assume that SB and PA may not be in direct opposition, even if they are typically contrasting. Stults-Kolehmainen et al. (2020) wrote that there may be restraining and propelling forces for both rest and movement acting simultaneously, and these might be modified or done flexibly based on needs and desires.

Related to light PA, we found that when the need for Physical Activity increased, the amount of light PA decreased. People with an increased need for Physical Activity are typically more interested in vigorous physical exercise that includes sweating and are more effective for growing muscles, staying fit, and being more physical (Reiss 1998, 2004, 2008).

In our research, the need for Tranquillity was linked to a higher amount of light PA. Light PA includes, for example, slow-tempo walking, gardening, and yoga. Therefore, people with a high need for Tranquillity may want to reduce their anxiety by adding light, relaxing, and low-risk exercise to their life to seek higher emotional calm. Also, according to Reiss (2008) and Reiss et al. (1986), people with a high need for Tranquillity desire to avoid experiencing anxiety or pain because they may be highly sensitive to pain. Therefore, they seek feelings of relaxation, whereas frustration may produce fear, anxiety, worry, and different symptoms of pain and uneasiness in their body. When they exercise, they also typically prefer to move in a way that does not hurt or produce any physical pain. That kind of moving is generally light and calm, as mentioned above. Also, Cremeans-Smith (2018) found that fear of pain was significantly linked with thoughts anticipating pain predicting participants’ exercise frequency. In their findings, men with a fear of pain reported more days with PA.

When the teaching experience increased, the amount of light PA increased. We assume that the longer experience the teachers have, the more they reduce stress by adding light PA. It may also be that the more experienced the teachers are, the more they move around in the classroom to support different learners individually. This phenomenon may be related to the higher age of experienced teachers. It is also possible that the intensity of physical exercise typically decreases with age. In addition, taking care of Family members was related to a lower amount of light PA.

In the RMP, the need for Physical Activity varies from a low need to a high need. Most people, about 60%, tend to score with a “balanced” or moderate need for Physical Activity (Reiss and Havercamp 1998). In this study, when the Family motive was higher, the moderate PA decreased. This may be explained by the time taken to take care of and be with family, which is time away from moderate PA. Perhaps these people prioritize Family over moving, and they would need to have a high need for Physical Activity to keep moving (and then most likely vigorously).

The more teaching experience people had, the higher the amount of moderate PA. On the other hand, a stable workplace was connected with a lower amount of moderate PA. Moreover, women had a higher amount of moderate PA than men, which could be explained by women in general having a somewhat higher need for Acceptance and a lower need for Physical Activity than men (Aflleje et al. 2022).

People with a high need for Physical Activity in the RMP prefer to move for the sake of moving. They typically enjoy hard and tough exercising, using their muscles, sweating, and different kinds of vigorous sports. The stronger the motive, the more different kinds of sports and physical activities these people tend to have as part of their lives, often daily. They may describe themselves as energetic, sporty, and fit (Reiss 2008; Mayor and Risku 2015). Therefore, it was not surprising that, compared with the FB results, the higher the need for Physical Activity, the higher the amount of vigorous PA during the measurement period.

On the other hand, if the respondents had a high need for Acceptance, the amount of vigorous PA was lower. People with a high need for Acceptance often feel more insecure and have a stronger fear of failure than those with a lower need for Acceptance (Reiss 2008). Vigorous PA is associated with higher risk-taking, which may be easier for those with a lower need for approval from others or who think they will succeed in anything; common thinking for those with a low need for Acceptance. Further, as Ball et al. (2014) discussed, motives for vigorous PA might be identified as related to competition, but also the enjoyment of exercise itself as the top motive.

Participants with a high need for Vengeance or winning also had more vigorous PA. A high need for Vengeance is associated with a need to win, battle, retaliate, not give up easily and be persistent (Reiss 2008). Top athletes benefit from the high need for Vengeance by tapping into the winning “spark” during decisive moments in competitions. It may also help them train more and harder than those with a lower need for Vengeance and winning. Often people who exercise a lot and hard are motivated by high Physical Activity and high Vengeance. Logically, this motive combination drives people to move. When doing sports, they are likely to enjoy competitive sports and measure their sports performance compared to their peers.

### Strength and limitations

To our knowledge, no earlier studies report the relationship between RMP and FB measurements. This study has made an important addition to the literature by examining the effects of motives on device-measured stress, recovery, sleep, and SB and PA. Our findings support the earlier evidence for the importance of the role of motives in PA promotion, specifically in vigorous PA. As a new observation, we found that the higher Acceptance motive was related to the lower amount of vigorous PA. It was surprising because, based on everyday experience, people exercise vigorously to get acceptance from others. The study also provides direction for future studies on the effects of motives on stress and recovery.

However, as a limitation of the study, it is partly unclear how motives are linked to the autonomic nervous system, and thus, stress, recovery, and sleep. The explanatory power of our model showed that several factors are needed to explain these links. In this study, we used only measured data. In the future, it would give us further information on this phenomenon to use qualitative data as well. Also, further research on the RMP and the 16 basic desires would significantly deepen the understanding of human motivation in general.

### Conclusions

We found that the 16 basic desires (motives) by Steven Reiss partly explained FB-measured stress, recovery, and sleep. The strongest links were found with sedentary behavior and physical activity. RMP motives such as Acceptance, Eating, Family, Physical Activity, Power, Tranquillity, and Vengeance were found to have a relationship with FB-measured outcomes. The Physical Activity motive appeared as the most significant one influencing stress, recovery, light PA, and vigorous PA. In conclusion, the basic desires explained sedentary and PA behavior better than the functioning of the autonomic nervous system. Finally, the 16 life motives can serve as a valuable theory for understanding better how to encourage people to have healthier habits.

## Data Availability

The datasets generated and analyzed during the current study are not publicly available because permission has not been applied for from either the participants or the Sustainable Brain Health project but are available from the corresponding author upon reasonable request.
